# ASSISTED CYCLING IN NON-AMBULANT ADULTS WITH CEREBRAL PALSY: EXPLORATORY EFFECTS AND FEASIBILITY IN RESIDENTIAL CARE

**DOI:** 10.2340/jrm.v58.45792

**Published:** 2026-09-22

**Authors:** Nanna S. POULSEN, Sara FALKENBERG, Noémie RECEVEUR, Sophia V. FRØLICH, Helle HÜCHE LARSEN, John VISSING

**Affiliations:** 1 Copenhagen Neuromuscular Center, Department of Neurology, Rigshospitalet, Copenhagen University Hospital, Copenhagen; 2 Jonstrupvang Bebyggelsen, Værløse; 3 Department of Neuroscience, University of Copenhagen, Copenhagen, Denmark

**Keywords:** exercise, cerebral palsy, wheelchairs, constipation, pain

## Abstract

**Objective:**

Adults with cerebral palsy (Gross Motor Function Classification System, GMFCS, levels III–V) have severely impaired motor function, causing fatigue, pain, constipation, and sleep disturbance, reducing life quality. Whether standard exercise is feasible or beneficial in this population is unknown, as spasticity, low muscle mass, and cognitive challenges may limit participation. Unable to engage in conventional exercise, they require tailored modalities. This study investigated whether assisted cycling is feasible and could improve key symptoms.

**Methods:**

14 adults with cerebral palsy (GMFCS III-V) completed a 10-week control period and 10 weeks of supervised, assisted cycling (3 x 20 min/week). Assessments at baseline, post-control, and post-exercise included key symptoms, feasibility, Gross Motor Function Measure-88, and a timed 1-km cycle test (time, power output, cadence).

**Results:**

Median attendance was 24/30 sessions. Heart rate increased during exercise (median 25.5 bpm). No significant group-level changes were observed, but individual improvements in pain, constipation, and power output were observed in some. Two participants experienced moderate adverse events affecting daily living.

**Conclusion:**

Assisted cycling is feasible and generally well tolerated in non-ambulant adults with cerebral palsy. While no group-level effects were detected, exploratory individual improvements in symptoms and cycling performance were observed in some, supporting further research on tailored exercise.

## LAY ABSTRACT

People with cerebral palsy are born with a brain injury. Those most severely affected often use a wheelchair, might not communicate with words, and can have learning difficulties. Many also experience tiredness, pain, constipation, poor sleep, and reduced life quality. Exercise might help, but many cannot perform regular exercise due to movement difficulties. In this study, 14 adults with severe cerebral palsy did a 10-week programme using an assisted exercise bike. They trained in group-based sessions for 20 min, 3 times/week, with support from staff. All completed the programme and most tolerated it well. While not everyone improved, some experienced less pain or constipation or became stronger. These findings suggest that assisted cycling can be a practical and acceptable way to support physical activity in adults with severe cerebral palsy. It may help improve comfort and physical function in daily life and could be used in residential care and rehabilitation settings.

Cerebral palsy (CP) is the most common childhood-onset physical disability, resulting from non-progressive brain injury in the perinatal period or early infancy (1). CP affects posture and motor control, and may also involve intellectual disability, epilepsy, sensory deficits, or impaired speech (1). The Gross Motor Function Classification System (GMFCS) classifies CP from level I (minimal impairment) to level V (most severe). Individuals classified as GMFCS levels III–V require part- or full-time wheelchair assistance.

People with CP commonly experience fatigue (50–60%) (2), pain (75%) (3), constipation (74%) (4), and sleep disorders (20%) (5), reducing quality of life (QoL) (6). These problems become more common with age (7) and higher GMFCS levels (8–10), leaving non-ambulant adults particularly affected.

Exercise has been shown to improve pain (11), constipation (12), and QoL (13) in non-disabled populations, suggesting similar benefits may be possible in adults with CP. However, motor control difficulties, low muscle mass, and cognitive impairments may limit participation and exercise responses. Although adults comprise approximately 80% of the CP population (6) and around 30% are non-ambulant (3), they are rarely included in research (6, 14). Consequently, only a few exercise studies in non-ambulant adults exists, mostly focusing on motor outcomes (15–18); very few have examined fatigue (19), pain (19), constipation (20), sleep (21), and QoL (22), and only in study populations with varying age and motor ability, with inconsistent findings.

Exercise modalities requiring minimal supervision may facilitate physical activity in daily life or care settings. Assisted cycle ergometers, which can support movement and compensate for low muscle mass, are promising because they allow participation with limited staff support.

This study explores whether assisted cycle exercise is feasible and can improve fatigue, lower back and leg pain, constipation, sleep quality, and QoL in non-ambulant adults with CP. We hypothesized that the intervention would be feasible and improve at least some outcomes.

## MATERIAL AND METHODS

### Participants

Participants were recruited from a 24-h care home in Denmark in October 2023. Inclusion criteria: age ≥ 18 years, clinical diagnosis of CP, and wheelchair use (manual or electric). Exclusion criteria: inability to use a cycle ergometer (e.g., due to contractures, inability to maintain a cycling position, or conditions preventing safe use), contraindications to exercise, ongoing psychiatric treatment (to avoid influencing QoL), > 2 h weekly exercise, inability to give informed consent, pregnancy, or inability to commit to 3 weekly sessions.

Convenience sampling was performed by care staff, followed by discussion with investigators. Written information was animated for comprehension and reviewed by caregivers. Cognitive function was not formally assessed but based on clinical observation.

### Study design

**Fig. 1:**
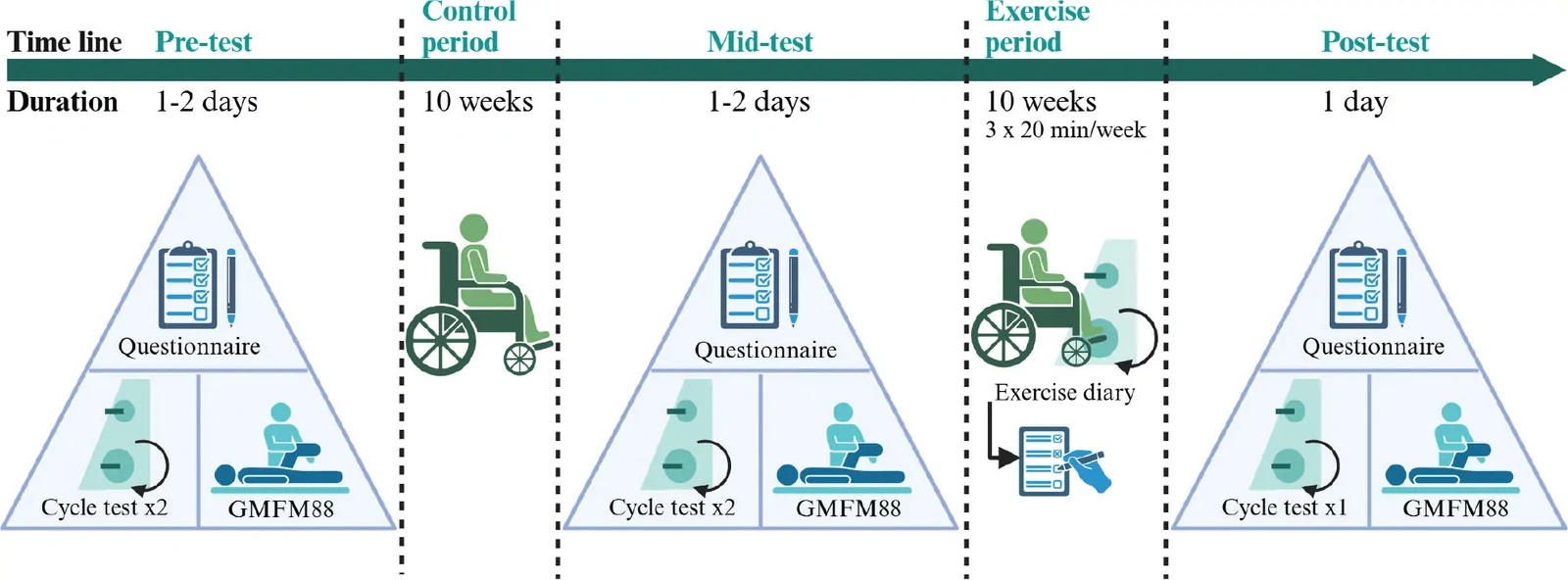
Study design. A 10-week control period was followed by a 10-week exercise period. Questionnaires, a timed 1-km assisted cycle test, and the gross motor function measure 88 was performed before the control period, between the 2 periods, and after the exercise period. Created at https://BioRender.com.

This self-controlled, exploratory, non-blinded, crossover study included a 10-week control period followed by 10 weeks of assisted, supervised cycling (Fig. 1). Testing occurred at baseline (pre-tests), between periods (mid-tests), and after exercise (post-tests). Participants started both periods simultaneously and were asked not to change pre-study physical activities or therapy appointments. Information on physical activity and therapy participation was recorded at each assessment and reviewed weekly during the intervention.

### Exercise intervention

Assisted, supervised, video-supported leg cycling was performed on a cycle ergometer (THERA-Trainer Tigo [THERA-Trainer, Hochdorf, Germany], Teramed A/S, leg-configuration only) in a group-based setting, 3 times weekly for 20 min, at the care home. Three weekly sessions reflected what could realistically be integrated into participants’ schedules, while 20-min sessions were considered a pragmatic duration likely to be tolerated. If leg cycling was impossible, assisted arm cycling (MOTOmed Reck; RECK Technik, Betzenweiler, Germany) was used. Participants exercised from a wheelchair or chair, with feet strapped to the pedals; calf supports or elastic thigh straps were used when needed. Motor-assisted pedalling was set at 25 rpm; extra load was added for stronger participants. Active effort was defined as the difference between motor-assisted rpm and participant-generated rpm. Watts, kilometres (km), and kilocalories (kcal) were displayed. Participants followed 2 virtual cycling routes from BoBo Pro 2.0 (Bo&Bo Ltd, Rosh Haayin, Israel) for motivation.

*Supervision:* Group-based sessions included 3–7 participants; individual sessions occurred if needed. Supervision by care staff (pedagogues) and investigators (medical doctor, NSP, or occupational therapist, SF) ensured motivation, safety, and correct execution, and logged kcal and km and whether the exercise was executed as intended.

*Progression:* Session frequency and duration remained unchanged to maintain a predictable routine. Limited individual progression was achieved via adding load, based on observed performance and tolerance, and encouraging maximal effort, through verbal encouragement, music, and virtual cycling routes.

*Monitoring:* Heart rate and perceived exertion were not used to guide intensity due to low muscle mass, cognitive, and communication limitations.

### Study outcomes

All tests were conducted and scored by the same rater (NSP). Outcomes reflected multiple ICF domains, including body functions (pain, fatigue, constipation), activity (motor function), and participation (QoL).

*Primary exploratory outcomes:* changes in fatigue, lower back and leg pain, constipation, sleep quality, and QoL. A positive exercise effect was defined as more outcomes improving than worsening, with at least 1 outcome improving.

- *Pain:* The Danish version of the Lower Back Pain Rating Scale (LBPRS, pain section) was used. Three items each for back and leg pain were scored on a Likert scale from 1–10 (total score range 3–30 per location). A visual analogue (VAS) scale was used for simplicity.
- *Constipation:* A questionnaire based on the Rome IV constipation criteria was used (Table SI). Total score range 0–7, ≥ 2 indicating constipation.
- *Sleep:* The Danish Pittsburgh Sleep Quality Index (PSQI) was used (23). Ten items were scored to assess 7 domains. Total score > 5 indicates poor sleep quality.
- *Fatigue:* Fatigue was assessed using an adapted Danish version of the Fatigue Severity Scale (FSS). Nine items were scored from 1–7. Higher scores indicate greater fatigue.
- *QoL:* QoL was assessed using an adapted Danish version of the short WHO Quality of Life questionnaire (WHOQOL-Bref). It consists of 26 items scored from 1–5. Four domains were derived: D1: physical health, D2: psychological health, D3: social relationships, and D4: environment. Higher scores indicate higher QoL. Question 9 in the Danish translation was corrected according to published recommendations (24).

Questionnaires were conducted through interviews due to reading and writing difficulties. Communication aids (iPads, communication boards, body language) were used when needed. Participants were asked directly with caregiver support to enhance autonomy and reduce proxy bias (25). To facilitate comprehension and participation among individuals with intellectual impairments, items from the FSS, WHOQOL-BREF, and questions 5–10 in PSQI were translated into easy-to-understand language, and Likert scales simplified and subsequently graded (questionnaires and scoring: Appendix S1). This approach was intended to support direct self-report participation and is consistent with previous studies reporting improved understanding when cognitive accessibility is enhanced (26).

*Secondary outcomes:* Secondary outcomes included feasibility measures, physiological and performance-based outcomes, and motor function.

**-** *Feasibility outcomes:* Attendance, completion rates (attending at least 1 session in the final week), adherence, and satisfaction.
**-** *Cycle test performance outcomes:* Participants familiarized themselves with the ergometer and selected their preferred resistance, which was kept constant across tests. With the motor set at 25 rpm, they biked 1 km as fast as possible. Power output (watts), cadence (rpm), and HR were measured continuously; HR with a pulse watch and only at mid- and post-tests to simplify the pre-tests. A passive test lasted 08:07 min. Outcomes were changes in time-to-completion, maximal power output, maximal cadence, cardiovascular response (HR), and maximal HR per maximal watt.
**-** *The GMFM-88:* The gross motor function assessed through 88 tasks scored 0–3, grouped into 5 dimensions: A: lying and rolling, B: sitting, C: crawling and kneeling, D: standing, and E: walking, running, and jumping. Higher sum scores indicate better function.

### Adverse events and caregiver reports

Adverse events to exercise were monitored and registered inspired by the ExHarm study (27). They were registered during each session, weekly, and at post-tests by self-reports, observations, predefined questions on muscle cramps, fatigue, delayed onset muscle soreness, and open questions.

Participants were asked at post-test if they thought exercising had been good for them and if they wanted to continue. Each participant’s caregiver completed a custom-made questionnaire consisting of 6 questions that each could be rated on a scale from 1–5. The questions assessed the caregiver’s perception of the participant’s exercise response, to cover how well changes in questionnaires and patient observations were also experienced by the caregiver.

### Statistics

*Sample size*. Given the absence of prior data on assisted cycling in non-ambulant adults with CP using our outcomes, no formal sample size calculation was performed. A target sample size of approximately 15 participants was defined on feasibility considerations.

*Descriptive statistics.* Descriptive statistics were calculated in R v.4.3.2 and Rstudio v.2024.04.2 (R Foundation for Statistical Computing, Vienna, Astrial). Packages: dplyr, rstatix, tidyverse, Rmisc, ggplot2, and viridis. *p* < 0.05 was considered statistically significant. All analyses should be considered exploratory. Data are presented as mean and 95% CI or median and bootstrapped 95% CI. Group-level and individual effects were investigated.

For analyses of changes in patient-reported outcomes, only participants with symptoms at baseline (score > 0) were included to reduce floor effect. Participants without baseline symptoms were described separately to assess whether symptom worsening occurred during the intervention.

Changes between all time-points were analysed within each outcome domain (3 pairwise comparisons: pre-mid, mid-post, pre-post). Ordinal outcomes (constipation and fatigue) were tested with Friedman’s test; changes in other efficacy outcomes, cycle-test outcomes, and GMFM-88 were analysed with repeated measures ANOVA when assumptions were met and Friedman’s test when not met. *Post hoc* pairwise comparisons (pre-mid, mid-post, pre-post) were performed using paired *t*-test or pairwise Wilcoxon signed-rank tests as appropriate, with Hochberg correction applied within each outcome domain. Sleep was first analysed in domains with no significance found, hence only the total score is reported. Statistical significance was based on adjusted *p*-values within each outcome domain; unadjusted *p*-values are reported for transparency only. Heart rate was analysed using paired *t*-tests (assumptions fulfilled), whereas HR/power output was analysed using pairwise Wilcoxon signed-rank tests between mid- and post-test.

Missing data were handled by pairwise deletion. A linear mixed model using maximum likelihood showed similar results; hence the simpler tests were used due to the small sample size. Participants were included in the analysis regardless of adherence to exercise.

### Ethical statement and registrations

The study followed the Helsinki Declaration of 1964. Written informed consent was obtained from all participants. Approval was granted by the Committee on Health Research Ethics of the Capital Region of Denmark (H-20075272).

The study is registered at ClinicalTrials.gov (NCT04677010).

## RESULTS

### Participants and time schedule

**Fig. 2:**
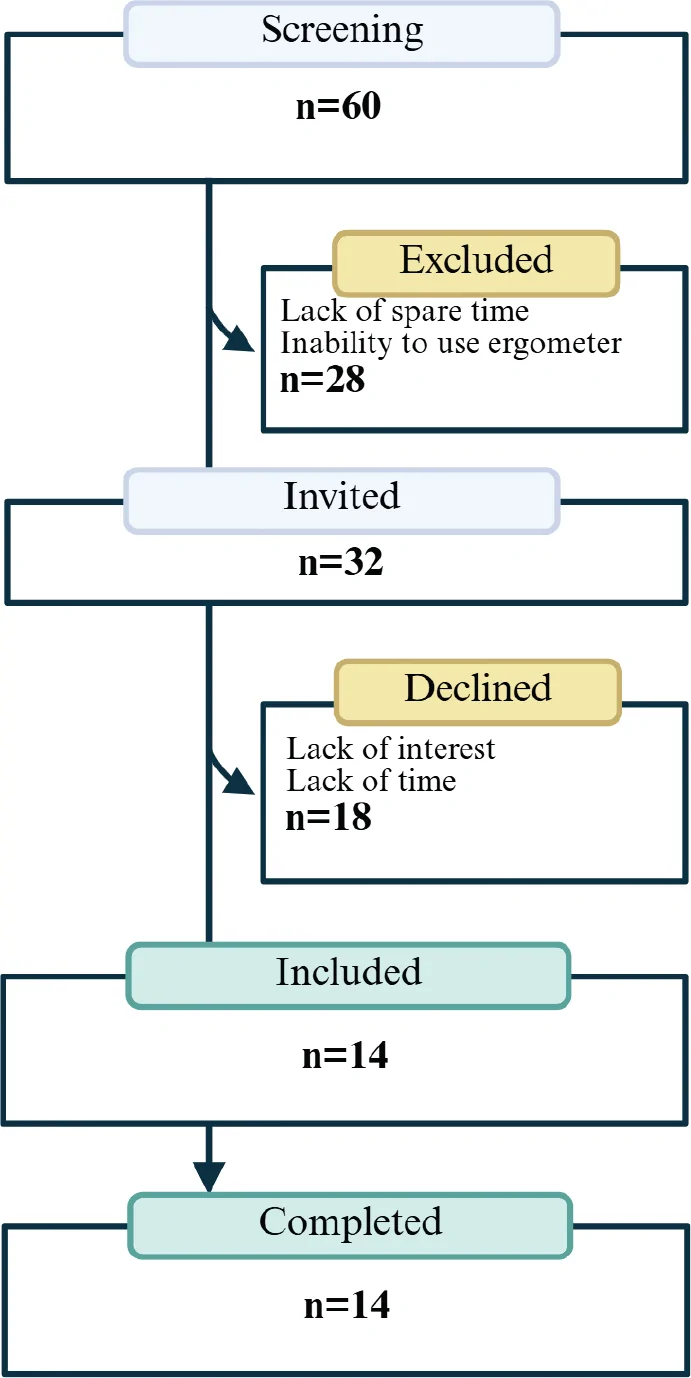
Flow diagram. Participant flow diagram showing the numbers screened, excluded (with reasons), declined (with reasons), included, and completing the study.

The flow of participants is illustrated in Fig. 2. Of 60 people living at or attending the care home for activities, 14 were included. Participants represented 5 care homes: 8 lived at the care home where the study took place, whereas 6 lived elsewhere but attended the care home for work and activities.

All 14 included participants had quadriplegic CP, mostly spastic, with variable residual voluntary movement **(**characteristics are presented in Table I). They were wheelchair users (GMFCS levels III–V), sedentary, and exhibited variable communication and cognitive function, including reduced attention, concentration, multitasking, organization, and planning.

**Table I:** Participant characteristics at baseline

Participant characteristics at baseline	*n* = 14
Median age, years (range)	45.5 (27–81)
Sex (M/F)	10/4
Topographic classification	
Quadriplegic spastic	12 (86%)
Quadriplegic ataxic	2 (14%)
Gross Motor Function Classification System Level	
III	5 (36%)
IV	6 (43%)
V	3 (21%)
Communication Function Classification System Level	
II	4 (29%)
III	4 (29%)
IV	6 (43%)
Type of wheelchair	
Manual	5 (36%)
Electric	9 (64%)
Baseline symptom prevalence	
Constipation	8 (57%)
Leg pain	8 (57%)
Lower back pain	3 (21%)
Fatigue	6 (43%)
Poor sleep quality	5 (36%)
Maximal power output during cycling	
< 5 watts	4 (29%)
5–20 watts	6 (43%)
> 20 watts	4 (29%)
Mid-test, median watts (range)	14.5 (2–86)
Post-test, median watts (range)	20.5 (2–90)
Maximal increase in HR during cycling (bpm)	
< 20	4 (29%)
20–40	8 (57%)
> 40	2 (14%)
% reached of calculated maximal HR	
50%	13 (93%)
60%	9 (64%)
70%	3 (21%)
Maximal HR during cycling	
Mid-test, median bpm (range)	105.5 (83–150)
Post-test, median bpm (range)	99 (84–143)
Maximal cadence during cycling	
Mid-test, median rpm (range)	47 (25–92)
Post-test, median rpm (range)	42.5 (25–95)
Time to finish 1 km	
Mid-test, median time in min (range)	5.0 (2.2–9.5)
Post-test, median time in min (range)	6.4 (2.1–17.3)

One participant used arm cycling, the rest did leg cycling. The exercise programme was adjusted for 2 participants by the investigators: 1 was reduced in frequency and duration from week 7 and switched to 10 min of high-intensity interval training (HIIT, alternating 1-min passive and high-intensity intervals) from week 9 due to adverse events. Another paused exercise for weeks 6–8 due to an unrelated knee injury. Sensitivity analyses excluding both participants yielded similar results; therefore, both participants were retained in the final analyses.

Post-tests occurred within 2 weeks after intervention. All participants received physiotherapy, 1–2 sessions per week, and none reported changes in leisure-time physical activities or therapy participation during the study.

### Feasibility

The participants reported high satisfaction: 12/14 participants (86%) found cycling beneficial, and 11 (79%) wanted to continue. One participant was not asked due to an oversight on the test day. Reasons for not wanting to continue were adverse events (1 participant) and lack of effects (1 participant).

Median attendance was 24/30 sessions (80%, range 17–28), with absences due to illness, leisure plans, adverse events (1 participant), or injury (1 participant). All completed the programme. Participants adhered well to the frequency and duration of exercise, but intensity varied, likely due to concentration and motor control challenges.

Most showed brief uneven pedalling, and a few intermittent pauses, especially those with lower GMFM-88 scores. Pauses happened mostly briefly a few times per exercise session but could vary up to 30 s per frequent stop. At 1 session, a participant covered only 200 m in 20 min. These issues could be eased by adjusting sitting position or reducing cognitive demands; reducing motor rpm had little or no effect. Total distance biked was 64 km (range 17–168). Caregivers rated exercise as beneficial for all but two participants (those with adverse events).

### Effect of exercise on pain, constipation, fatigue, sleep, and quality of life

Baseline symptom prevalence is indicated in Table I.

Sample size varied across analyses of exploratory patient-reported outcomes. Participants without baseline symptoms were not included in analyses of change. Additional exclusions were made because of initiation of medical treatment for constipation (*n* = 2), absence of pain perception (*n* = 1, excluded from pain analyses), or arm cycling (*n* = 1, excluded from leg pain analysis). In addition, 5 isolated questionnaire assessments were missing (constipation *n* = 1; sleep *n* = 2; fatigue *n* = 1, QoL *n* = 1).

Participants without baseline symptoms were analysed descriptively to assess potential worsening; only two showed slight increases in pain or constipation scores.

**Fig. 3:**
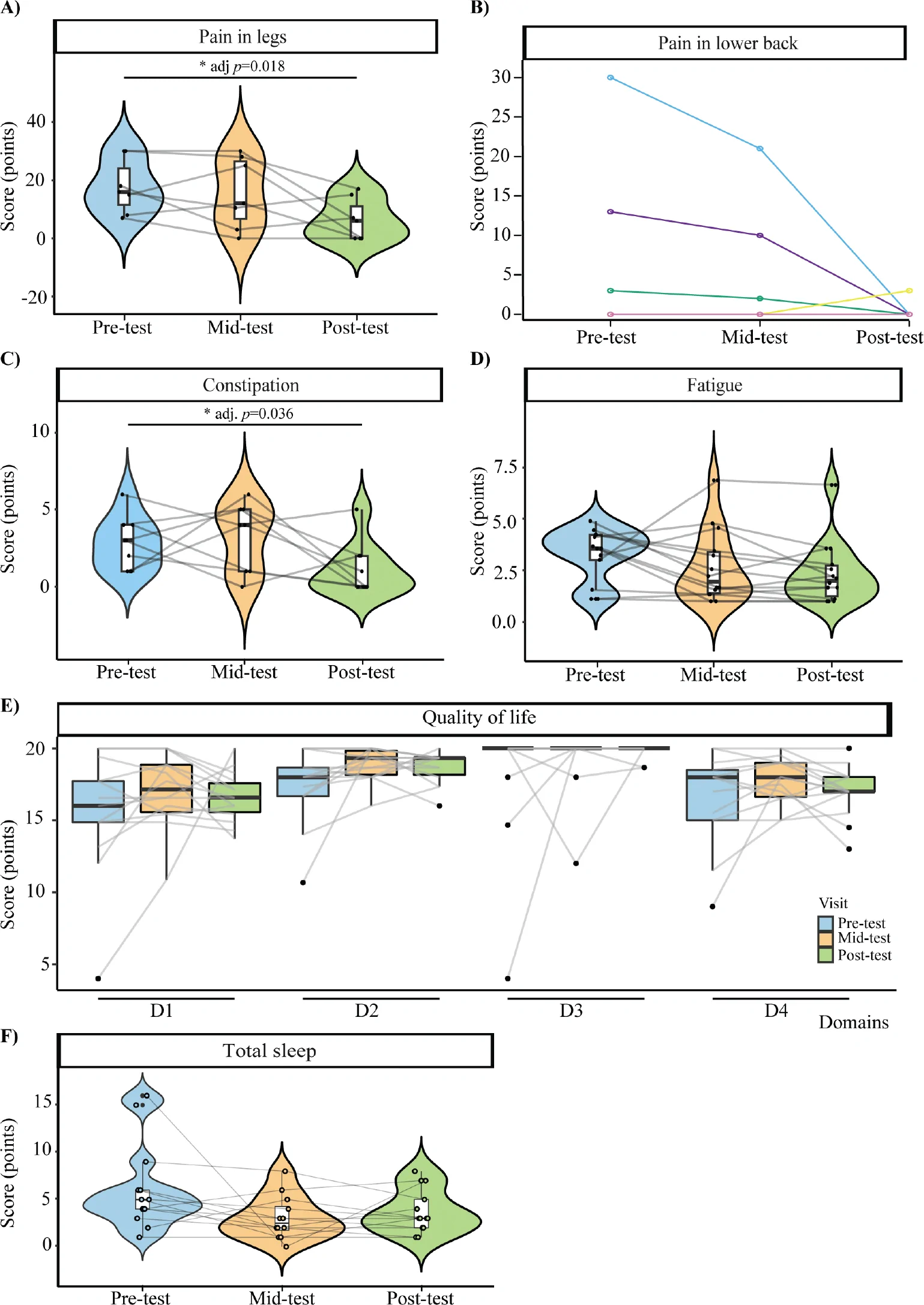
Effects of assisted cycle exercise on efficacy outcomes. Individual participant trajectories with overlaid violin- and boxplots illustrate changes with assisted cycle exercise. The control period is between pre- and mid-test, the exercise period between mid- and post-test. Changes are shown for (A) pain in legs (*n* = 7), (B) pain in lower back (shown as a spaghetti plot, *n* = 14), (C) constipation (*n* = 9), (D) fatigue (*n* = 13), (E) quality of life (*n* = 14), and (F) sleep quality (*n* = 13). Significant differences are shown with adjusted *p*-values; 95% confidence intervals and *p*-values for all comparisons, including non-significant comparisons, are presented in Table SII. Subdomains of QOL are labelled as D1–D4: D1: physical health, D2: psychological health, D3: social relationships, and D4: environment. Asterisks (*) indicate statistically significant differences (adjusted *p*<0.05).

Leg pain and constipation scores were reduced from pre- to post-test, but not from mid- to post-test (Fig. 3A and C; Table SII). Pain-related improvement was supported by reduced analgesic use in 2 participants and caregiver observations. Constipation scores improved in 6 of the 9 analysed participants, with fewer participants meeting diagnostic criteria after the intervention (3 vs 6).

Lower back pain was not statistically analysed due to limited sample size (*n* = 3), although all participants showed lower scores after the intervention (Fig. 3B).

No statistically significant differences were observed in fatigue scores, sleep quality scores, or QoL scores after correction for multiple testing (Fig. 3D–F; Table SII).

### Effect of exercise on motor function

**Fig. 4:**
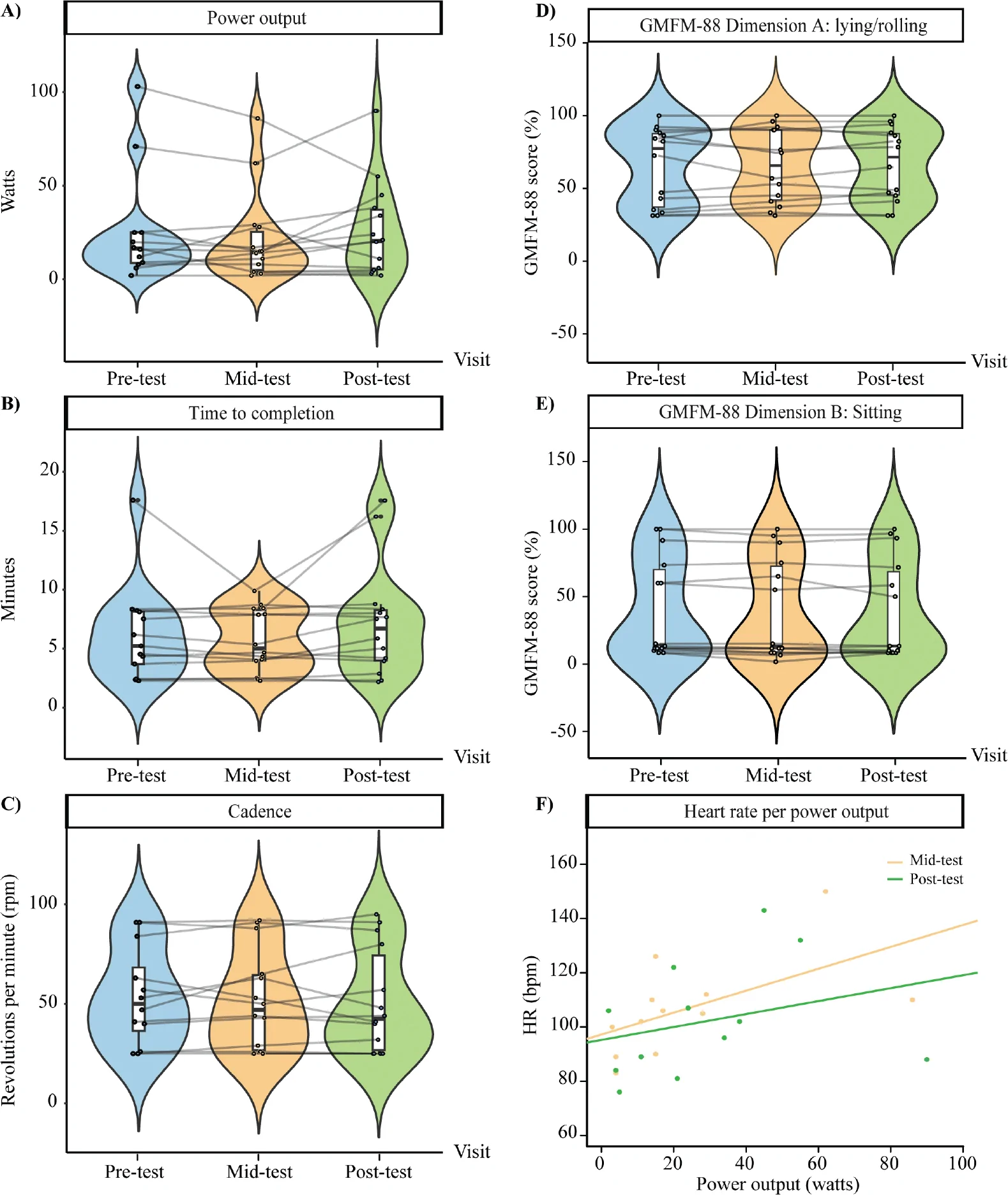
Effects of assisted cycle exercise on motor function and heart rate per watt. The individual participants’ trajectories with overlaid violin- and boxplots illustrate changes with assisted cycle exercise in (A) maximum power output (*n* = 14), (B) time to bike 1 km (*n* = 14), (C) maximum cadence (*n* = 14), (D) Gross Motor Function measure 88 Dimension A (*n* = 14), and (E) Gross Motor Function measure 88 Dimension B (*n* = 14). The control period is between pre- and mid-test, the exercise period between mid- and post-test. HR was not measured at pre-test. Heart rate per power output is shown in (F) with individual data points and regression lines for mid- and post-test; used for descriptive purposes only (*n* = 12). Bpm: beats per minute; GMFM-88: gross motor function measure 88 HR: heart rate; rpm: revolutions per minute.

Changes in outcomes from the cycle test were analysed with and without the 2 participants with many adverse events (the 2 strongest participants in Fig. 4A). Conclusions were unchanged; therefore, results are presented including all participants.

No significant changes were observed in maximal power output (see Fig. 4A; Table SII). Despite this, individual responses varied, with half of the participants showing increases in power output, including marked improvements in a subset of participants. The largest improvements were observed primarily among participants classified as GMFCS level III, although a few participants classified as GMFCS level IV also improved.

No changes were observed in time to complete 1 km, maximal cadence, or GMFM-88 dimension A (Fig. 4B–E; Table SII). The estimate of GMFM-88 dimension B indicated a small decrease, although the confidence interval included zero. GMFM-88 dimensions C, D, and E were completed by 6, 3, and 2 participants, respectively, and were therefore not analysed statistically. Scores in these dimensions remained largely unchanged at the individual level.

### Cardiovascular response to exercise

HR increased during a cycle test in all participants (median mid-test increase: 25.5 bpm, range: 10–91). Most (93%) reached ≥ 50% of estimated maximal HR but only a few (21%) reached ≥ 70%.

There was no statistically significant difference in HR/power output from mid- to post-test (see Table SII), although bootstrap confidence intervals did not include zero. Likewise, the regression line appeared lower at post-test (Fig. 4F), but this visual trend was descriptive only and not formally tested.

### Adverse events

Most events were mild, transient, and frequency decreased during the intervention (delayed-onset muscle soreness, fatigue, cramps, Table II). Fatigue was generally mild, but 2 participants experienced moderate fatigue; symptoms resolved in 1 but persisted in the other.

**Table II:** Adverse events

Event	Participants (of 14) *n* (%)	Severity	Impact on intervention	Duration
Delayed onset muscle soreness	9 (64)	Mild	None	First few days after exercise; frequency reduces for 8, but starts at the end of the exercise period for 1 (doing arm cycle exercise)
Fatigue	9 (64)	Mild for 7, moderate for 2	None for 8, change in exercise time and intensity for 1	Until next day; frequency reduces during the exercise period for 8, but for 1 remains unchanged until change to interval training
Muscle cramps	5 (36)	Mild–moderate	None	Few minutes; mainly at beginning of exercise period
Wound	2 (14)	Mild–moderate	Foam attachments to the cycle steering for 1, changes in seat position for 1	A few days until healing
Mood fluctuations	1 (7)	Mild–moderate	None	Worst on exercise days, lasting throughout all 10 weeks
Decreased stability during transfers	1 (7)	Mild–moderate	None	Shortly after exercise

Two unexpected adverse events occurred: 1 knee abrasion from friction from the chair while biking, and 1 participant with uncontrolled movements injured his hand on the steering. Both resolved by adjustments.

## DISCUSSION

This exploratory study indicates that assisted, group-based cycling was feasible and well tolerated for most non-ambulant adults with CP. Participants reported high satisfaction, demonstrated a cardiovascular response to exercise, and some showed exploratory, hypothesis-generating, individual improvements in pain, constipation, and power output.

### Effects of assisted cycle exercise

Non-ambulant adults with CP frequently experience pain, constipation, fatigue, sleep disturbances, and reduced QoL, partly due to sedentary lifestyles, reduced circulation, and impaired bowel motility. Although no group-level effects were detected, several participants experienced lower pain and constipation scores following the intervention. These were supported by self-reports, caregiver observations, and reduced pain medication. Such findings align with prior recommendations for multimodal pain assessment in individuals with cognitive impairments (28). One previous study reported increased proximal lower limb pain following longer (1-h), strength-based sessions (19), suggesting that the shorter, assisted cycling intervention in our study may mitigate such adverse events.

The lack of group-level effects is likely multifactorial. First, heterogeneity in motor function, cognitive abilities, and comorbidities may influence responsiveness to exercise. Second, our sample size limits statistical power. Third, ceiling effects may have reduced sensitivity, particularly for QoL, where participants’ scores were comparable or even exceeding Danish population norms (29). Similar challenges in detecting QoL changes have been reported in exercise studies with ambulant people with CP, and likely reflect a combination of participant heterogeneity, intervention variability, short durations, small samples, and questionnaire limitations (30, 31). Finally, while most exercise studies in individuals with CP classified as GMFCS levels I–III last 6–12 weeks, longer interventions might be necessary for adults at GMFCS levels III–V due to lower motor performance, slower motor learning, and higher prevalence of intellectual impairments.

Sleep and fatigue did not change, unlike prior positive group or individual findings in ambulatory and non-ambulatory individuals with CP (19, 21). Factors such as questionnaire design, participant characteristics, and external influences likely contributed to the lacking effect in our study. Further, sleep and fatigue are multifactorial, influenced by care routines, noise, medication side effects, and emotional stress, and might require multimodal interventions.

### Cardiovascular and physical outcomes

All participants increased HR during exercise, with most reaching levels consistent with moderate cardiovascular effort – a notable increase from a sedentary baseline, likely beneficial for cardiovascular health (32).

Although no significant group-level improvements were observed in power output, half of the participants improved, with 3 nearly doubling their output, suggesting strength gains. Participants with higher motor function responded better, likely reflecting their ability to generate higher exercise intensity. Concentration and motor challenges might have limited performance in some individuals, contributing to response variability. These observations highlight the importance of individualized assessment and the limitations of timed maximal exercise tests in this population, where maintaining focus and consistent effort can be challenging.

No significant change occurred in GMFM-88 scores. Previous studies reporting improvements often involved younger participants, different intervention types, or broader GMFCS ranges (15–18).

### Exercise challenges

While most participants enjoyed the exercise, uneven pedalling and adverse events were noted.

Uneven pedalling occurred in some participants, with a few having occasional pauses in rotation, sometimes limiting distance covered. This was more pronounced in individuals with lower motor function and might be explained by several mechanisms:

1. *Motor performance and learning* rely on motor control, cognition, and visual and proprioceptive feedback. Cerebral injury, muscle atrophy, altered sensory inputs, and visual impairments reduce performance and complicate learning. Cognitive-motor multitasking can be difficult, especially for individuals with CP (33), worsening pedalling consistency. This could explain the uneven cycling and worsening issues under higher cognitive demands. With practice, motor performance will likely improve, but the learning process might be slow or variable.
2. *Muscle contractures, passive stiffness, and corrective surgeries* (e.g., tendon elongations) likely alter the muscle force–length relation, reducing force generation near joint extremes (34). This might explain why uneven pedalling often occurred on leg extension and why slight position changes could sometimes improve performance.
3. *Involuntary sustained or intermittent contractions* like spasticity, spastic dystonia, and dystonia are common in adults with CP (35). The prevalence increases with lower motor function (35) and symptoms worsen with focused attention to the movement (36).This might explain the sudden pedalling pauses and the worsening when attention was drawn to it. Studies suggest that exercise does not worsen spasticity and may improve functional outcomes over time (15, 37).

Adverse events were mostly mild and resolved with continued exercise, although 2 out of 14 participants experienced moderate fatigue affecting daily life. Both maintained relatively high pedalling speeds, and 1 displayed involuntary movements that may have increased energy expenditure. While these events highlight that assisted cycling may not be equally tolerated by all individuals with severe CP, they should be considered alongside the high adherence, participant satisfaction, and absence of severe adverse events observed in this study.

Overall, assisted cycling was well tolerated for most participants despite occasional pauses in rotation and 2 moderate fatigue-related adverse events. Therefore, the potential benefits of assisted cycling should be balanced against the risk of fatigue and variable exercise performance. Careful monitoring, individualized progression, and adjustments such as breaks, positional changes, variable intensity, and close supervision appeared important for supporting tolerability and participation. Such adaptations may be particularly important for individuals classified as GMFCS level V, who frequently experienced pauses in rotation, and for those with involuntary movements or higher power output, who may have higher energy demands.

### Questionnaires for individuals with intellectual impairments

Patient-reported outcomes in adults with CP and intellectual impairments are often completed by caregivers or proxies (28, 38). However, direct engagement enhances participants’ sense of being heard and respected (38) and reduces systematic bias or mismatch between the caregiver’s perception and the participant’s experience (25). Consequently, developing and using questionnaires adapted for individuals with intellectual impairments is important, and such tools are slowly emerging, particularly in pain assessment (28, 39).

In this study, all participants were able to contribute to patient-reported outcome measures when supported by caregiver support and guided interview techniques. For 1 participant with more severe intellectual impairment, responses could not be reliably graded, and a simplified yes/no/unknown format was used for fatigue and QoL. Although these measures were not formally validated, the findings suggest that direct self-report may be feasible in this cohort when appropriate support is provided.

Based on this experience, questionnaire administration in this population may benefit from simplified language, concrete examples, a focus on present experiences, repeated measures over time, and systematic checking of understanding during completion.

These observations demonstrate both the challenges and the potential of involving adults with CP and intellectual impairments in self-reported outcome assessments. Future work should focus on developing and validating accessible outcome measures for adults with CP and intellectual impairments.

### Limitations

Investigating adults with severe motor and cognitive impairments presents inherent challenges. The small sample size and clinical heterogeneity of CP, including the wide age range (27–81 years), variability in motor control, and cognitive function, reflect real-world characteristics of non-ambulant adults with CP, but limit statistical power and generalizability. This heterogeneity may have contributed to variable exercise responses.

Another limitation relates to the assessment of patient-reported outcomes. The questionnaires were not validated in adults with severe CP and intellectual impairments, and the FSS and WHOQOL-BREF were adapted into easy-to-understand language. Although considered necessary to facilitate participation, these adaptations have not undergone formal validation. Consequently, self-reported outcomes should be interpreted with caution and regarded as exploratory.

Finally, several methodological limitations should be acknowledged. Outcome assessors were not blinded to the intervention, introducing a risk of detection bias. Convenience sampling might have caused selection bias. Group-based training, chosen for logistical reasons, may have additionally supported mood and energy through social interaction.

### Conclusion

This exploratory study indicates that assisted group-based cycling is feasible and generally well tolerated in non-ambulant adults with CP, although a few experienced moderate fatigue. With tailored adjustments and supervision, assisted cycling may be feasible to implement in residential or rehabilitation settings. While no consistent group-level effects were observed, some participants showed improvements in pain, constipation, and cycling power. These findings are hypothesis-generating and support further investigation of individualized exercise interventions to optimize effectiveness, minimize adverse events, and tailor exercise to participants’ functional and cognitive abilities.

## Supplementary Material




