# Supplementary material for: ASSISTED CYCLING IN NON-AMBULANT ADULTS WITH CEREBRAL PALSY: EXPLORATORY EFFECTS AND FEASIBILITY IN RESIDENTIAL CARE
Source: J Rehabil Med. 2026 Sep 22;58:45792. doi: 10.2340/jrm.v58.45792 (PMC13613323; doi:10.2340/jrm.v58.45792)
Supplement: Supplementary file 1 [file JRM-58-45792-s1.pdf]

Table SI. Custom-made questionnaire to assess constipation.

Adapted from the ROME-IV criteria. Translated from Danish. Each "yes" gives one point, and a sum score ≥2 indicates constipation.

The last month…:	Yes	No
I had two or fewer defaecations per week		
It was often difficult to get evacuated entirely		
I had lumpy or hard stools many times		
I often had a sensation of incomplete evacuation		
I had rarely loose stools without the use of medication		
I had to manually assist the tools out		
I often felt that my rectum was blocked		

Table SII. Changes in questionnaires, motor function and HR/power output

	Pre-mid changes		Pre-post changes		Mid-post changes	
	Change (95%CI)	*p*-value	Change (95%CI)	*p*-value	Change (95%CI)	*p*-value
**Questionnaires**						
Pain score, leg (n=7)	Mean -2.21 (-9.67 – 5.24)	Raw: 0.495 Adj: 0.495	Mean -11.29 (-17.98 – -4.60)	Raw: **0.006** Adj: **0.018**	Mean -9.07 (-20.56 – 2.42)	Raw: 0.102 Adj: 0.204
Constipation score (n=9)	Median: 0 (-3 – 3)	Raw: 0.944 Adj: 0.944	Mean -1.00 (-4.00 – -1.00)	Raw: **0.012** Adj**: 0.036**	Mean -2.00 (-5.00 – 1.00)	Raw: 0.105 Adj: 0.210
Fatigue score (n=14)	Median -0.78 (-1.89 – 0.22)	Raw: 0.191 Adj: 0.197	Median -1.33 (-1.67 – -0.11)	Raw: **0.036** Adj: 0.107	Median -0.28 (-0.67 – 0.00)	Raw: 0.197 Adj: 0.197
Total sleep quality score (n=13)	Median -1 (-3.5 – -0.5)	Raw: 0.081 Adj: 0.162	Median -2.00 (-3.00 – -2.00)	Raw: **0.021** Adj: 0.063	Median 0.00 (-1.00 – 1.50)	Raw: 0.952 Adj: 0.952
QoL score, domain 1 (n=14)	Median 0.57 (0.00 – 2.86)	Raw: 0.097 Adj: 0.282	Median 0.00 (-1.71 – 2.29)	Raw: 0.937 Adj: 0.282	Median -0.86 (-2.29 – 0.00)	Raw: 0.141 Adj: 0.282
QoL score, domain 2 (n=14)	Median 0.67 (0.00 – 2.00)	Raw: **0.042** Adj: 0.127	Median 1.33 (-0.67 – 2.67)	Raw: 0.099 Adj: 0.198	Median -0.33 (-0.67 – 0.67)	Raw: 0.503 Adj: 0.503
QoL score, domain 3 (n=14)	Median 0.00 (0.00 – 0.00)	Raw: 0.684 Adj: 0.789	Median 0.00 (0.00 – 0.00)	Raw: 0.279 Adj: 0.789	Median 0.00 (0.00 – 0.00)	Raw: 0.789 Adj: 0.789
QoL score, domain 4 (n=14)	Median 0.50 (0.00 – 1.50)	Raw: 0.181 Adj: 0.360	Median 0.00 (-2.00 – 1.50)	Raw: 0.929 Adj: 0.929	Median 1.00 (-2.00 – 0.25)	Raw: 0.090 Adj: 0.271
**Motor function and HR/power output**						
Maximal power (watts, n=14)	Median -0.5 (-9.87 – 2.00)	Raw: 0.476 Adj: 0.476	Median: 8 (-1.91 – 16.00)	Raw: 0.195 Adj: 0.468	Median 3.50 (-1.50 – 9.20)	Raw: 0.234 Adj: 0.468
Time-to-complete (min, n=14)	Median 0.00 (-0.46 – 0.27)	Raw: 0.450 Adj: 0.450	Median 0.15 (-0.19 – 0.45)	Raw: 0.421 Adj: 0.45	Median 0.27 (-0.32 – 1.57)	Raw: 0.119 Adj: 0.357
Cadence (rpm, n=14)	Median 2.00 (-1.50 – 5.50)	Raw: 0.357 Adj: 0.929	Median 0.00 (-1.00 – 4.00)	Raw: 0.553 Adj: 0.929	Median 0.00 (-4.00 – 3.00)	Raw: 0.929 Adj: 0.929
GMFM-88, dimension A (n=14)	Median 0.00 (-1.96 – 1.96)	Raw: 0.858 Adj: 0.858	Median 0.98 (-1.96 – 3.92)	Raw: 0.722 Adj: 0.858	Median 0.00 (-1.96 – 3.92)	Raw: 0.284 Adj: 0.852
GMFM-88, dimension B (n=14)	Median -1.67 (-3.33 – 0.00)	Raw: 0.138 Adj: 0.276	Median -1.67 (-2.50 – 0.0)	Raw: **0.014** Adj: **0.043**	Median 0.00 (-1.67 – 2.50)	Raw: 1.000 Adj: 1.000
HR/power output (bpm/watt, n=12)	NA	NA	NA	NA	Median -1.77 (-5.32 – -0.47)	Raw: 0.063

NA: Not applicable due to lack of data collection at pre-test.

Adj, adjusted; GMFM-88, Gross motor function measure 88; HR, Heart rate; rpm, revolutions per minute; QoL, quality of life
