# Supplementary material for: ASSISTED CYCLING IN NON-AMBULANT ADULTS WITH CEREBRAL PALSY: EXPLORATORY EFFECTS AND FEASIBILITY IN RESIDENTIAL CARE
Source: J Rehabil Med. 2026 Sep 22;58:45792. doi: 10.2340/jrm.v58.45792 (PMC13613323; doi:10.2340/jrm.v58.45792)
Supplement: Supplementary file 2 [file JRM-58-45792-s2.pdf]

Appendix S1

Participants were asked directly, with caregivers supporting understanding but not providing answers. Gestures (hands close vs. apart) and simplified verbal cues were used to indicate gradations. Keywords used during pre-tests were documented and reused during subsequent tests to ensure consistency. This approach is consistent with previous studies showing that adults with mild-to-moderate intellectual impairments can provide valid self-reports when cognitive accessibility is enhanced[25]

.

Body language was used to indicate response gradation: hands close for "a little" and apart for "a lot", making the process more relatable.

Adapted Fatigue severity scoring scale

Questionnaire:

The rephrased questionnaire, translated below from Danish to English:

1) **Do you want to do less when you are tired?**
2) **Do you get tired when exercising?**
3) **Do you get tired quickly?**
4) **Are you sometimes so tired, that you cannot do what you usually do?**
5) **Are you sometimes so tired, that you get into trouble?**
6) **Are you sometimes so tired, that you cannot exercise for a longer time?**
7) **Are you sometimes so tired, that you cannot work properly?**
8) **Is tiredness one of the three things that bother you most?**
9) **Are you sometimes so tired, that it affects your family and leisure time?**

Scoring guide:

Each question should be answered with yes, no, or neither/nor. Continue with the following question:

For yes:

- Question 1-7 and 9: Does it happen sometimes (score of 5), often (score of 6), or always (score of 7)?

- Questions 8: Does it bother you a little (score of 5), somewhat (score of 6), or a lot (score of 7)?

For no:

- Question 1-7 and 9: Does it happen sometimes (score of 3), rarely (score of 2), or never (score of 1)?
- Question 8: Does it bother you a little (score of 3), somewhat (score of 2), or a lot (score of 1)?

For neither/nor:

- Score of 4

Adapted WHO-QOL-BREF

Questionnaire:

The rephrased questionnaire, translated below from Danish to English, with scoring for each question.

10) **Are you happy with your life?**
11) **Are you happy with your health?**
12) **Are you limited by pain?**
13) **Can you manage without medication?**
14) **Do you enjoy life?**
15) **Do you have a purposeful life?**
16) **Are you able to concentrate?**
17) **Do you feel safe?**
18) **Are you happy with what it looks like here [read: at the day care center] and where you live?**
19) **Do you have enough energy?**
20) **Are you happy with your look?**
21) **Do you have enough money?**
22) **Can you find answers to your questions?**
23) **Can you engage in leisure activities?**

24) Can you get around at the day care center and at home?

25) Are you happy with your sleep?

26) Can you do what you want to do in daily life?

27) Are you happy with how good you work?

28) Are you happy with yourself?

29) Are you happy with other people?

30) Are you happy with your sex life?

Question excluded

31) Are you happy with the help you get from your friends?

32) Are you happy with the place you live?

33) Can you visit a doctor if you need to?

34) Are you happy with your opportunities for transport?

35) Do you sometimes have bad feelings, like being sad or afraid?

Scoring guide:

Each question should be answered with yes, no, or neither/nor. For "neither/nor" a score of 3 should be given. Continue with:

- Q1-3, 5-9, 1, 19-20, 22-23:
  - For yes: a little (4) or a lot (5)? For no: not really (2) or not at all (1)?
- Q4, 10, 12-18, 24-26:
  - For yes: sometimes (4) or always (5)? For no: rarely (2) or never (1)?

Question 21 on sex life was removed since it was difficult for many to answer, and one had a significant emotional reaction to the question. It was replaced with the average of questions 20 and 22 (according to the scoring guide), as all belong to the same domain allowing comparison with other studies.
